# Peak I of the human auditory brainstem response results from the somatic regions of type I spiral ganglion cells: Evidence from computer modeling

**DOI:** 10.1016/j.heares.2014.07.001

**Published:** 2014-09

**Authors:** Frank Rattay, Simon M. Danner

**Affiliations:** a Institute for Analysis and Scientific Computing, TU Vienna, Vienna, Austria; b Center for Medical Physics and Biomedical Engineering, Medical University of Vienna, Austria

**Keywords:** ABR, auditory brainstem response, AP, action potential, CSF, cerebrospinal fluid, eABR, electrically evoked ABR, FE, finite element method, IAM, internal auditory meatus, IHC, inner hair cell, OHC, other hair cell, SGC, spiral ganglion cells

## Abstract

Early neural responses to acoustic signals can be electrically recorded as a series of waves, termed the auditory brainstem response (ABR). The latencies of the ABR waves are important for clinical and neurophysiological evaluations. Using a biophysical model of transmembrane currents along spiral ganglion cells, we show that in human (i) the non-myelinated somatic regions of type I cells, which innervate inner hair cells, predominantly contribute to peak I, (ii) the supra-strong postsynaptic stimulating current (400 pA) and transmembrane currents of the myelinated peripheral axons of type I cells are an order smaller; such postsynaptic currents correspond to the short latencies of a small recordable ABR peak I’, (iii) the ABR signal involvement of the central axon of bipolar type I cells is more effective than their peripheral counterpart as the doubled diameter causes larger transmembrane currents and a larger spike dipole-length, (iv) non-myelinated fibers of type II cells which innervate the outer hair cells generate essentially larger transmembrane currents but their ABR contribution is small because of the small ratio type II/type I cells, low firing rates and a short dipole length of spikes propagating slowly in non-myelinated fibers. Using a finite element model of a simplified head, peaks I_n_ and II (where I_n_ is the negative peak after peak I) are found to be stationary potentials when volleys of spikes cross the external electrical conductivity barrier at the bone&dura/CSF and at the CSF/brainstem interface whereas peaks I’ and I may be generated by strong local transmembrane currents as postsynaptic events at the distal ending and the soma region of type I cells, respectively. All simulated human inter-peak times (I–I′, II–I, I_n_–I) are close to published data.

## Highlights

• ABR peak I is generated by transmembrane currents from type I spiral ganglion cells.
• Maximum transmembrane currents in human type I cells are from the soma region.
• The maximum transmembrane currents generate ABR peak I.
• Peak I time is independent from the conductance of the extracellular media.
• Thus, peak I is not a stationary potential which is in contrast to peak II.

## 1 Introduction

Jewett and Williston (1971) recorded from the scalp of humans a series of seven quite consistent waves which are known as the auditory brainstem responses (ABR). As early response to acoustic clicks (<10 ms) these waves, labeled I–VII, are also found in animals, e.g. in cat (Jewett, 1970, Melcher et al., 1996), rat (Hall, 1990) and guinea pig (Brown and Patuzzi, 2010, Malherbe et al., 2013). The latencies of wave I–VII are of physiological and clinical interest (Moller et al., 1988, Mason and Herrmann, 1998), especially the electrically evoked ABR (eABR) in cochlear implantees (Abbas and Brown, 1991, Guiraud et al., 2007, Undurraga et al., 2013). Each ABR wave consists of a positive peak followed by a negative one called, e.g. P1 or peak I and N1 or I_n_.

Esteves et al. (2009) reported an ABR time difference between the peaks of wave I and wave III of 2.13 ± 0.14 ms (*n* = 120 ears) in normal hearing human subjects. It is generally accepted that waves I and II are caused by the spiral ganglion cells (SGC) of the auditory nerve that connect the hair cells in the cochlea with the next neural processing unit, the cochlear nucleus, where wave III is generated (Moller et al., 1988). Roughly speaking, the interpeak time III–I may correspond to the action potential (AP) conduction time from the hair cell to the cochlear nucleus. However, which functional segments of the SGCs produce peak I is still an open question.

Disagreements about peak I generators are consequences of different interpretations of the recorded potentials which result from transmembrane currents along the auditory nerve. From guinea pig experiments it was concluded that peak I originates from the SGC segment where APs are first produced (Matsushima, 1982). Other investigators found in humans a peak I′ preceding peak I. It is assumed that peak I′ is generated in the auditory nerve by the postsynaptic currents from hair cells (Hashimoto, 1982, Hughes and Fino, 1985, Moore et al., 1992, Davis-Gunter et al., 2001). According to another study on guinea pigs, peak I may be generated at a rather large distance along the SGC pathway (Brown and Patuzzi, 2010) when the conducted APs are passing the dura mater at the proximal end of the internal auditory meatus (IAM, a canal in the temporal bone for the passage of the cranial nerves VII and VIII).

**Fig. 1:**
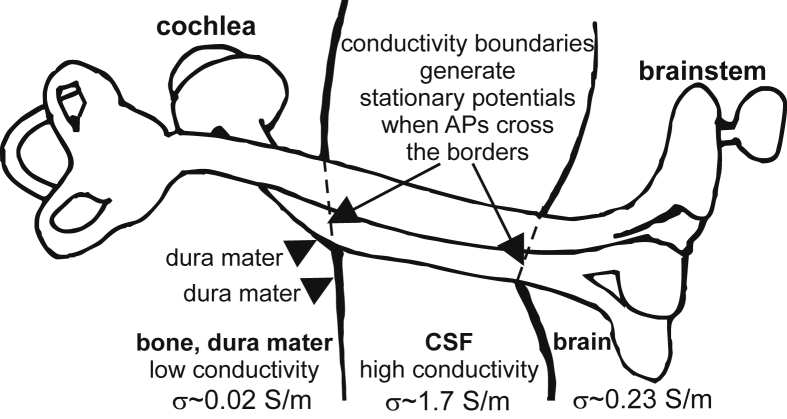
Sketch of the anatomy surrounding the auditory nerve. External electrical conductance along the auditory nerve changes (i) when it exits the internal auditory meatus (IAM, a canal in the temporal bone for the auditory nerve, the vestibular nerve and the facial nerve) into the subarachnoid space and (ii) when it subsequently enters the brainstem. The respective discrete conductance interfaces are between (temporal bone + dura mater)/CSF and CSF/brainstem (marked by dashed lines). The dura mater marked by arrowheads covers also the IAM (Lescanne et al., 2002) and due to its similar conductance to bone, together they electrically form a single macroscopic compartment. Redrawn after Martin et al. (1995) who compared the passage of the human auditory nerve from IAM to brain with and without CSF (air) and found evidence that the left border (left arrow) is the generator of the first negative ABR peak, I_n_, whereas the CSF/brain border causes ABR peak II.

Peak latencies can be similar or different for recording electrodes close to the current source (near field) and at greater distances (far field), e.g. in the brain case or on the scalp. An electrode in the vicinity of a single axon records a passing AP as a positive/negative/positive sequence of potentials as the axonal transmembrane current influx is separated by outflow in the leading and trailing part of the conducted spike. The three related (positive/negative/positive) near field peaks broaden and decrease quickly in amplitude when the electrode is moved away from the axon. In the far field a ‘stationary potential’ arises when many APs pass within a small time window a region where the conductance of the extracellular medium changes (Fig. 1; Nakanishi, 1982, Stegeman et al., 1987). Combining ABR data with the knowledge about stationary potentials lead to contradictory hypotheses: (i) peak I may be caused by the distal IAM ending (Tasaki et al., 1954), (ii) the low cerebrospinal fluid (CSF) conductance between the proximal IAM end and the higher conductance of the brainstem may cause two sequential peaks namely negative peak I_n_ (this is the next ABR minimum after peak I, which is also called N1) and peak II when APs enter brainstem (Martin et al., 1995), (iii) Brown and Patuzzi (2010) suggest that a single electrical barrier, the dura mater, produces both peak I and peak I_n_ when the leading respectively the trailing dipole of the conducted transmembrane currents encounter this barrier.

About 95% of SGCs are type I cells (Spoendlin, 1975), which are innervated by inner hair cells (IHC). In humans such a bipolar cell has a myelinated axon that is interrupted by a non-myelinated region around the soma. The non-myelinated somatic region is a human peculiarity, which is rarely found in cochleas of animals (Ota and Kimura, 1980, Nadol, 1988). Type II SGCs, innervated by outer hair cells (OHC), are non-myelinated, their axon and soma diameters are smaller and consequently signal conduction is slow (about 1/9) in comparison to type I cells (Rattay et al., 2013). Lack of insulation by internodes in axons of type II cells demands essentially stronger transmembrane currents compared to axons of type I cells. Concerning the origin of ABR peak I, this fact needs an investigation as the higher ion currents could compensate for the small numbers of type II cells.

**Fig. 2:**
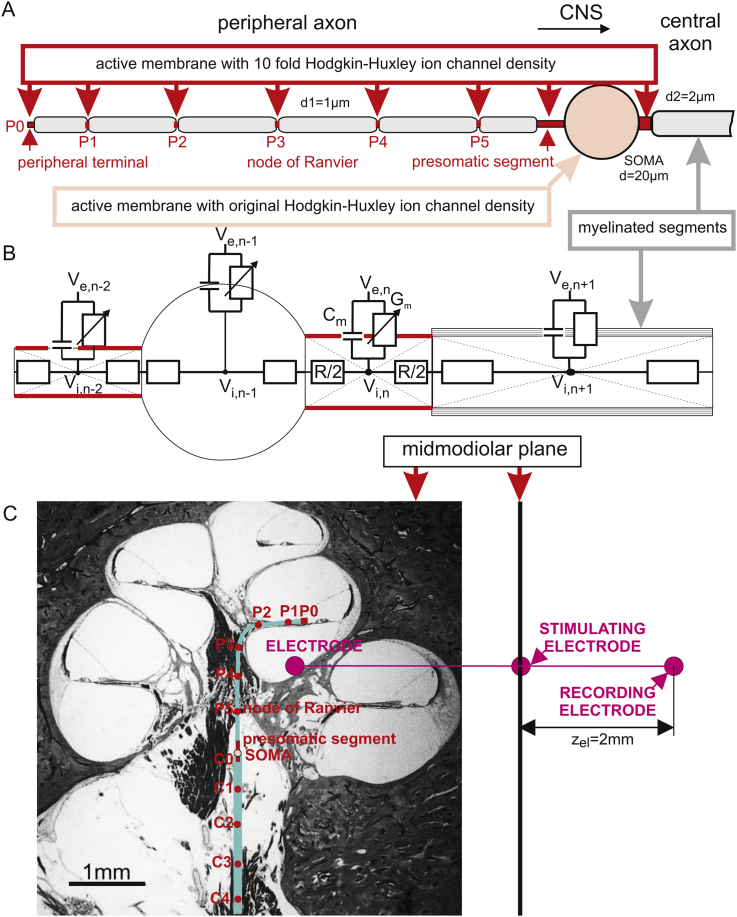
Compartment model of a mylinated spiral ganglion cell (SGC) and its position in the human cochlea. A: scheme of a type I cell. Myelinated segments are shown in gray. Excitable (active) membranes with high ion channel densities (red segments) in the peripheral terminal P0 and in the nodes of Ranvier are needed for spike amplification. B: Electrical network model. The currents are defined by extracellular potential *V*_*e*_, intracellular potential *V*_*i*_, membrane capacitance *C*_*m*_, membrane conductance *G*_*m*_ and intracellular resistance R. Natural excitation by synaptic current from a hair cell ribbon synapse is simulated as current injection into the first compartment (peripheral terminal P0). For natural excitation all extracellular potentials *V*_*e*_ are assumed to be zero. For nonmyelinated type II cells the same approach was used with uniform ion channel densities as in the original Hodgkin-Huxley model and with constant compartment lengths in the axons. C (left): Cross-section of a human cochlea with two dimensional pathway of the model neuron. Active compartments of the central axon are indicated as C0 (postsomatic segment), C1, C2, etc. The modeled SGC can be activated either by current injection in P0 or by currents from the stimulating electrode. C (right): Simulated neural activities are recorded at distance z_el_ from the midmodiolar plane.

Using a biophysical model (Fig. 2, Rattay et al., 2001a) we could show that spike conduction in mammalian SGCs is characterized by the following four phases: a postsynaptic delay, constant velocity in the peripheral axon, a presomatic delay and constant velocity in the central axon (Rattay et al., 2013). AP duration (330 μs) and axonal velocity of spike conduction were in good agreement with recorded mammalian peripheral nerve data. Although this modeling study did not include signal delays for the synaptic transfer in the cochlear nucleus, the 2.24 ms spike conduction time along the average human type I cell is already longer than the time differences between ABR peaks III and I as found by Esteves et al. (2009), indicating that peak I appears later than the first elicited activities in the auditory nerve. On the other hand our computational evaluation of the membrane currents demonstrated that surprisingly high postsynaptic currents from IHC ribbon synapses with amplitudes of 400 pA (Grant et al., 2010) outnumber threshold by a factor of at least 15 (Rattay et al., 2013).

The goal of the following investigation is to analyze whether such strong postsynaptic currents in the peripheral terminals of SGCs could generate ABR peak I or which other neural components could be the generators. Alternatively, peak I could be a stationary potential, e.g. generated when spikes conducted along the auditory nerve arrive at the left dashed line in Fig. 1.

## 2 Methods

### 2.1 Compartment model of SGCs

A compartment model of a human type I cell is described in detail in Rattay et al. (2001a). This model was used for intracellular and extracellular stimulation. According to Fig. 2A, the cell consists of segments with active membranes with high (marked red) and low ion channel density (pale pink). Additionally, there are myelinated internodes (gray) where the membrane is assumed to be passive which means membrane currents, which depend on voltage sensitive ion channel gating mechanisms, are negligible. The high ion channel density, modeled as 10-fold Hodgkin Huxley membrane conductance, is needed for signal amplification along the neural pathway. This sodium channel density is comparable to nodes of Ranvier in axons of the mammalian peripheral nerve system (Rattay and Aberham, 1993, Rattay et al., 2003). Beside the peripheral terminal and the nodes of Ranvier, the high sodium channel concentration is applied in the pre- and postsomatic compartment. According to the biophysical model approach, strong sodium current influx is especially needed in the presomatic region to load the large capacitance of the non-myelinated soma in order to conduct the spike across the soma into the central axon (Rattay et al., 2001a). The high sodium channel density in the presomatic region is also confirmed experimentally in non-myelinated type I cells of the mouse (Hossain et al., 2005).

Applying Kirchhoff's law (the sum of all currents is zero) to the n-th compartment of the electric network of Fig. 2B results in

(1) $$\frac{\mathrm{d}(V_{i,n}-V_{e,n})}{\mathrm{d}t}C_{m,n}+I_{\text{ion},n}+\frac{V_{i,n}-V_{i,n-1}}{R_{n}/2+R_{n-1}/2}+\frac{V_{i,n}-V_{i,n+1}}{R_{n}/2+R_{n+1}/2}=0\text{,}$$

where the first two terms are the transmembrane currents which are the postulated generator elements of the ABR signal. They correspond to capacitance current and ion current across the membrane whereas the next two terms describe the intracellular current flow to the left and right compartment. Introducing the reduced membrane voltage *V* = *V*_*i*_ − *V*_*e*_ − *V*_rest_ (*V*_*i*_ , *V*_*e*_ and *V*_rest_ are the intracellular, extracellular and resting potential, respectively) leads to the following system of differential equations (Rattay, 1999, Rattay et al., 2001a):

(2) $$\frac{\mathrm{d}V_{n}}{\mathrm{d}t}=\left[-I_{\text{ion},n}+\frac{V_{n-1}-V_{n}}{R_{n-1}/2+R_{n}/2}+\frac{V_{n+1}-V_{n}}{R_{n+1}/2+R_{n}/2}\right]/C_{m,n}+\left[\frac{V_{e,n-1}-V_{e,n}}{R_{n-1}/2+R_{n}/2}+\frac{V_{e,n+1}-V_{e,n}}{R_{n+1}/2+R_{n}/2}\right]/C_{m,n}\text{.}$$

For the first and last compartments Eqs. (1), (2)) have a reduced form because of lack of neighbors. The membrane surface *A*_*n*_ of every compartment has to be calculated to find *C*_*m*,*n*_ = *A*_*n*_·*c*_*m*,*n*_ (*c*_*m*,*n*_ is the specific membrane capacitance; note that the capacitance of *N* layers of membranes is proportional to 1/*N*).

The ion membrane current is governed by the gating mechanisms of specific voltage sensitive ion channels. It consists of two components

(3) $$I_{\text{ion }}=A_{n}\cdot i_{\text{ion},n}+I_{\text{noise},n}\text{,}$$

where *i*_ion_ is the ionic membrane current density and *I*_noise,*n*_ represents ion channel current fluctuations in active compartments. The effective noise current is assumed to be proportional to the square root of the number of sodium channels within a compartment (for details see Rattay et al., 2001a).

In compartments with passive membranes (internodes) the term *I*_*ion*_ of Eqs. (1), (2)) is a current with constant conductance *I*_ion_ = *g*_*m*_*A*_*n*_*V*_*n*_/*N*, where membrane conductance *g*_*m*_ is 1 mS/cm² and the number of insulating layers of cell membranes *N* is 40 and 80 for the peripheral and central axon, respectively (Rattay et al., 2001a).

Using this method the temporal profiles of the transmembrane voltage and the transmembrane currents of each compartment were computed. The ion currents of active membranes were simulated with original Hodgkin Huxley kinetics (Hodgkin and Huxley, 1952) at 27 °C. At this temperature AP duration fits recorded data and, moreover, the model is appropriate for the non-myelinated regions of SGCs (Hartmann et al., 1984, Motz and Rattay, 1986, Rattay et al., 2013). Further supports for this approach are found in the subsections ‘proof of concept’ and in the discussion section.

### 2.2 Stimulation of SGCs

Current injection into the first compartment P0 of the modeled neurons simulated the synaptic activation by hair cells. The shape and amplitude of the postsynaptic currents of type I cells were defined by an average amplitude *I*_0_ of 400 pA, 280 μs for time to peak and a decay time constant *τ* of 370 μs as recorded in adult P20 and P60 rats (Grant et al., 2010). The same shape but with smaller amplitude *I*_*0*_ of 26 pA was used for the stimulation of type II cells corresponding to signal strengths recorded by Weisz et al. (2012).

**Fig. 3:**
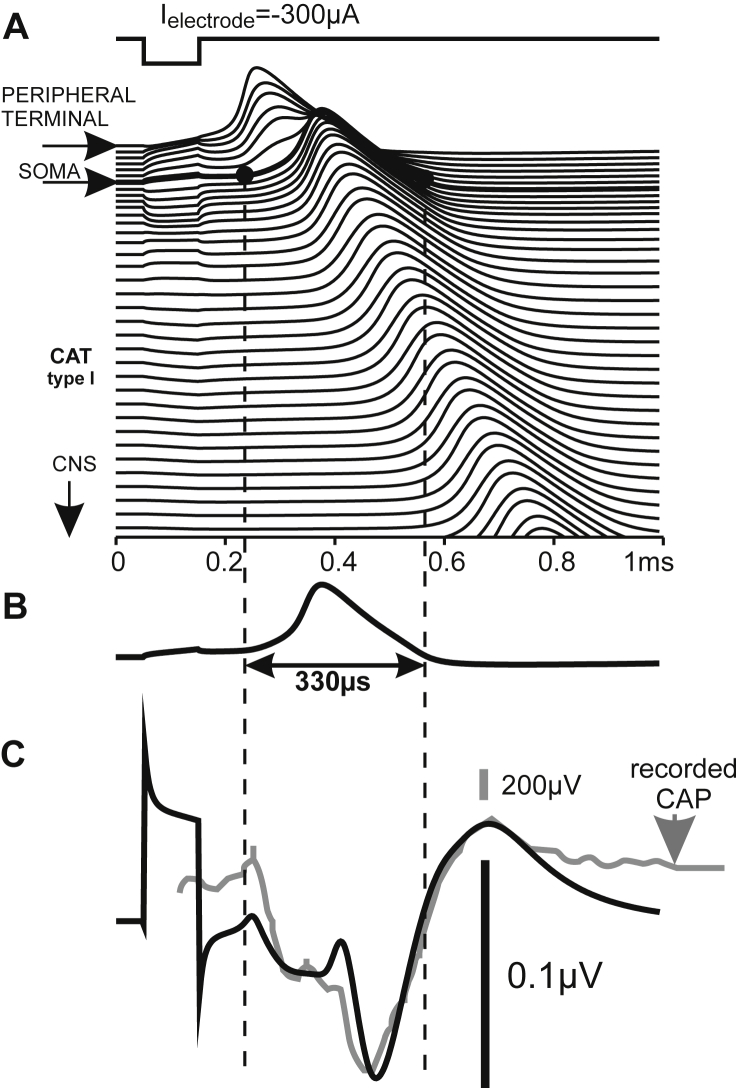
Temporal profiles of transmembrane voltages and extracellular potentials of an extracellularly stimulated feline type I cell. A small spherical electrode simulates monopolar cathodic stimulation with a cochlear implant. A: The transmembrane voltage lines are shifted vertically according to their distance along the neural path. During application of the 100 μs stimulus pulse the steady state voltage across the membrane is disturbed in every compartment. For the selected electrode placement the disturbance is strongest in the peripheral terminal and spike initiation is similar here to natural signaling. B: The short spike duration is demonstrated with the redrawn transmembrane voltage of the presomatic compartment. C: Simulated extracellular potential (black) and experimentally recorded (gray) intracochlear voltage profiles generated with a cochlear implant show similar temporal characteristics although the simulated single cell activity is compared with a compound action potential (CAP) recording. The gray curve is a redrawn version of Fig. 1 (intracochlear recording, cathodic pulse −11.1 dB rel. 1 mA) from Miller et al. (2004). Simulated situation corresponds to feline morphometry (short peripheral axon, myelinated soma) as described in Rattay et al. (2013).

When a cell is stimulated externally, the extracellular potentials cause virtual injected currents in every compartment (Rattay, 1999, 2003). In contrast to synaptic stimulation, the differing distances of the compartments to the stimulating electrode (Fig. 2C) cause strong variations in the individual driving forces. An example of the resulting membrane voltage variations along the neural path is seen in the upstroke–downstroke–upstroke–downstroke sequence during the application of the stimulus pulse in Fig. 3A.

In order to calculate the extracellular potentials *V*_*e*_, needed in Eq. (2), we assumed an infinite homogeneous extracellular medium. Ignoring capacitance effects of tissue, the extracellular potential was calculated as

(4) $$V_{e}=\rho_{e}\cdot I_{\text{electrode}}/4\pi r,$$

where *r* is the center–center distance between a compartment of interest and the spherical electrode. *ρ*_*e*_ = 300 Ohm cm (Rattay et al., 2001a) was assumed to be the mean resistivity of the extracellular medium.

### 2.3 A simple approach for the contribution of a single SGC to the ABR signal

Within an infinite homogeneous extracellular medium Eq. (4) describes the relationship between the current of a point source and the potential in its surrounding. Using Eq. (4) together with the superposition principle, a first approach for the *V*_ABR_ signal contribution of a single activated SGC was simulated as the sum of the contributions of both transmembrane current components from all compartments

(5) $$V_{\text{ABR}}=\frac{\rho_{e}}{4\pi }\sum_{n=1}^{\text{DIM}}\left(I_{\text{ion},n}+I_{c,n}\right)/r_{n}\text{.}$$

As explained above, for every compartment the transmembrane current has a resistive component *I*_ion_ and a capacitance component *I*_*c*_ = *C*_*m*_·d*V/*d*t*, which is the first term in Eq. (1). The standard position of the recording electrode is shifted 2 mm from the stimulating electrode in orthogonal direction to the midmodiolar plane (Fig. 2C). DIM is the number of compartments used. Standard compartment structure of the 32 mm long model neuron was the following: P0, internode, P1, internode, …, P5, internode, 3 presomatic compartments, soma (compartment 16), postsomatic compartment C0, internode, central node C1, 59 internode-node combinations, internode, resulting in DIM = 135 compartments.

### 2.4 Proof of concept

As proof of the method we simulated an intracochlear recording of an electrically stimulated feline cochlea. The temporal similarity of the computed and recorded signal (Fig. 3C) demonstrates the suitability of the simple membrane current model.

### 2.5 A simplified human head model

**Fig. 4:**
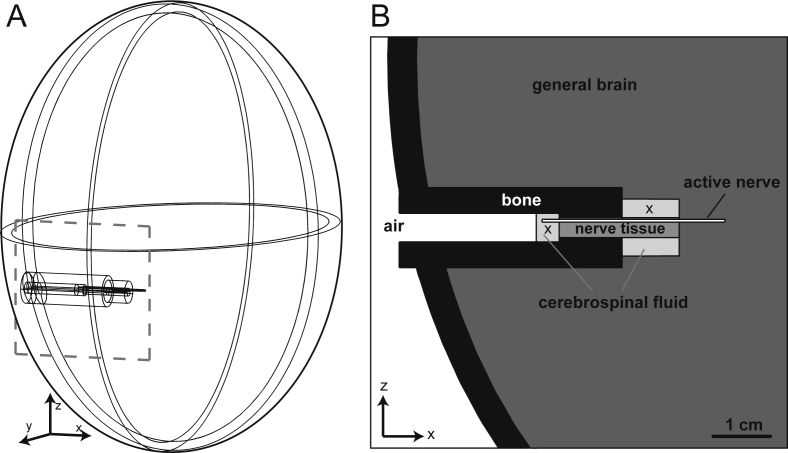
Head model to simulate the electric field generated by transmembrane currents in the auditory nerve. A: The axes of the elliptic skull (7 mm thick) are 7, 10 and 10 cm in x, *y* and *z* direction, respectively. Cylindrical structures in *x*-direction represent the simplified geometry of the cochlea, the auditory nerve and the surrounding conductive media with specific conductances denoted by gray shading in the zoomed part B. The transmembrane currents of a population of 1000 synchronously spiking type I cells (1000 fold currents of a single cell) are the input data for the finite element calculation. These ABR generating currents from the active subpopulation of the auditory nerve were assumed to flow in orthogonal direction from the surface of a cylinder referred as active nerve. Positions of recording electrodes are marked by X.

In order to explain how the polarity of measured signals depends on the recording position and for the analysis of other far field effects, we used a simple elliptic closed head model where all current paths were closed within the head. The model consisted of the skull, the ear canal, the cochlea, the internal auditory meatus, the cerebrospinal fluid, the brain and the auditory nerve itself (Fig. 4). Using the finite element method (FE), we calculated the electric field for selected recording positions when 1000 identical SGCs were synchronously activated. For this purpose part of the auditory nerve was modeled as an active cylinder with 0.5 mm diameter and 32 mm length (denoted as active nerve in Fig. 4B). The surface of the cylinder acted as a time dependent current source summarizing the transmembrane currents of 1000 active SGCs within the corresponding volume.

In more detail, using COMSOL Multiphysics 4.4 for Mac OS X 10.9.2 (COMSOL Inc., Burlington, MA, USA), the generated electrical potential Φ in the volume conductor was calculated by a reduced form of Maxwell's equation

(6) ∇·(σ∇Φ)=0,

which depended on the conductivity *σ* of the medium. Neumann type boundary conditions were applied,

(7) (σ∇Φ)·n=f(x,t),

where *n* was the normal vector and *f*(*x*,*t*) a predefined time and location depending function of current density. For all external surfaces the normal current density was restricted to zero (i.e., *f*(*x*,*t*) = 0 for all *x* and *t*). Along the surface of the active cylinder *f*(*x*,*t*) represented the transmembrane currents of 1000 synchronously firing SGCs.

The calculation was performed by an iterative time discrete solver for 2.5 ms in time steps of 0.025 ms. Conductivities (shown as graded gray values in Fig. 4B) were: bone 0.02 S/m, brain (as gray matter) 0.23 S/m, nerve fibers 0.6 S/m (longitudinal) and 0.083 S/m (transversal), and cerebrospinal fluid 1.7 S/m (Rattay et al., 2000, Danner et al., 2011).

## 3 Results

### 3.1 Type I cells respond with a short delay to strong synaptic stimuli

**Fig. 5:**
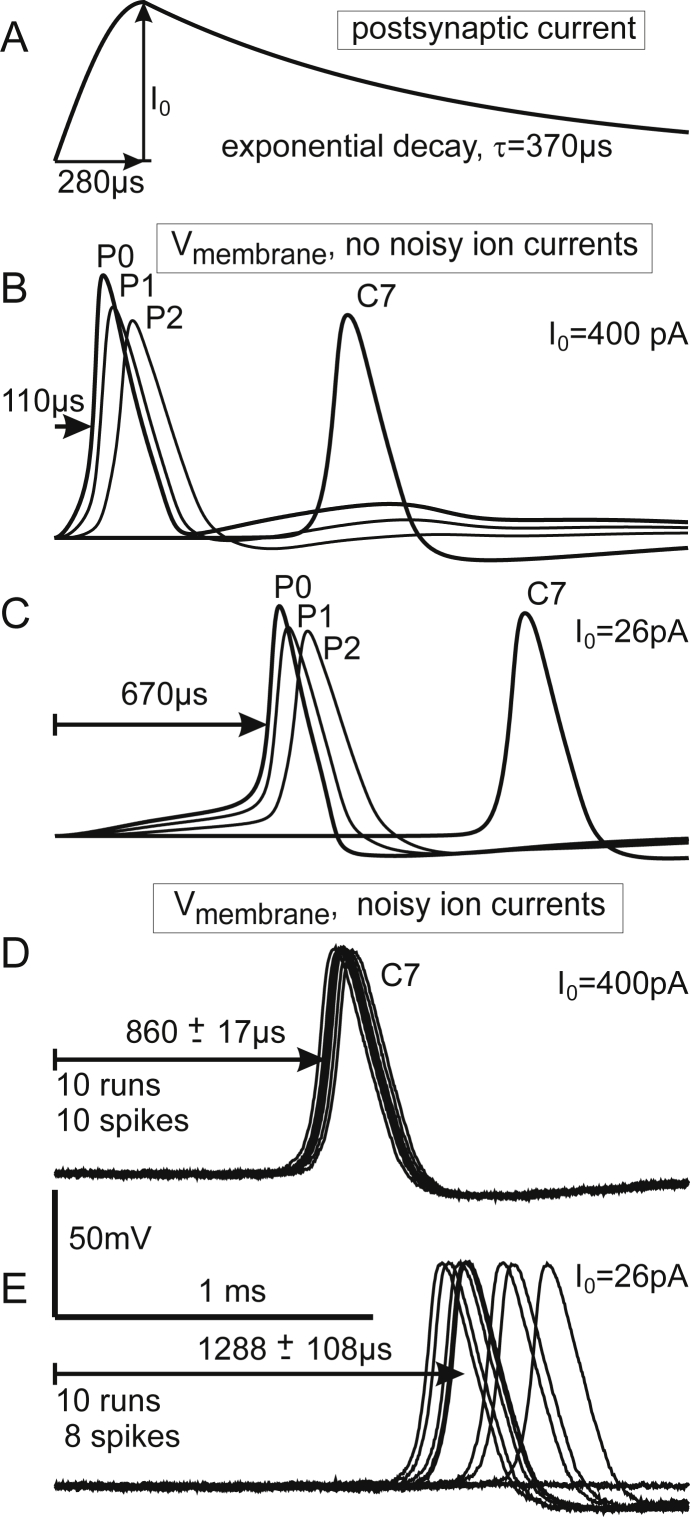
Responses of a type I cell to strong and weak synaptic stimuli. The active membrane is simulated without and with ion current fluctuations. A: Postsynaptic current as recorded in rat type I cells (Grant et al., 2010). B: Transmembrane voltage profiles at selected compartments for *I*_0_ = 400 pA. C: Reduction of stimulus amplitude defers spike initiation. D: Including ion current fluctuation causes small jitter for strong stimulation, e.g. in central node C7. E: Decrease of stimulus intensity causes fewer spikes and a significantly increased jitter at C7. Simulated with standard cell I data of Fig. 1: *d*1 = 1 μm, *d*2 = 2 μm (peripheral and central axon diameters), soma diameter 20 μm.

The first part of the rising AP in the peripheral terminal P0 is closely related to the stimulus intensity. This property is documented in Fig. 5. Synaptic SGC excitation was simulated by a 400 pA current injection in P0. 280 μs rise time and exponential decay with a time constant of 370 μs (Fig. 5A) were the same as observed in rat type I cells. This strong postsynaptic current caused a short AP delay (110 μs, Fig. 5B) whereas a drastically weaker synaptic stimulation (26 pA, Fig. 5C) had a delay of 670 μs. In order to show the influence of stimulus strength, we applied this 26 pA stimulus intensity again to a type I cell, although it was the mean postsynaptic current recorded in rat type II cells (Weisz et al., 2012). When ion current fluctuations are included, the model approach confirms the two important advantages of strong stimuli from inner hair cell ribbon synapses (Fig. 5D and E): a short AP delay and small jitter characterize quick and accurate signaling from IHCs to the cochlear nucleus.

### 3.2 Contribution of transmembrane currents to the extracellular potential

**Fig. 6:**
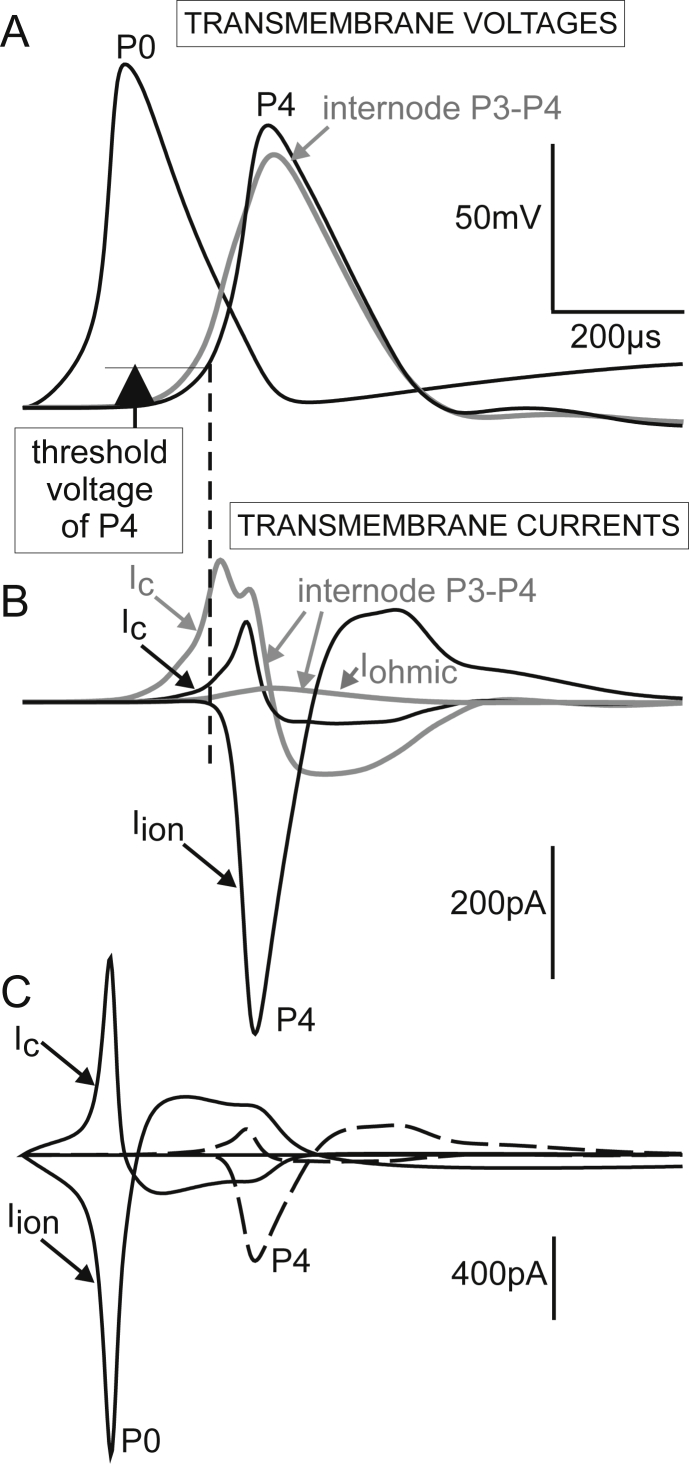
Voltages and currents across the membranes of selected compartments of a type I cell. A: In comparison to a typical node of Ranvier (P4) membrane voltages *V* are larger in the strongly stimulated compartment P0 but smaller in the internode (gray). B: The ion current of P4 has a larger delay than the capacitance current of P4 as ion gates do not open before threshold voltage is reached (marked by vertical dashed line). C: Comparison of membrane currents in the terminal P0 and node of Ranvier P4 (broken lines) demonstrates the larger P0 currents as a consequence of the larger terminal surface.

Due to the strong stimulus, the AP peak at P0 of a type I cell was 21.7% larger than the conducted membrane voltage peak, e.g. at node P4 (Fig. 6A). The transmembrane current amplitudes at a node or internode in the peripheral axon were roughly comparable to that of the 400 pA stimulus (Fig. 6B). However, both transmembrane currents, *I*_ion_ and *I*_*c*_, at P0 exceeded that at P4 because of the bigger surface area of the active membrane (Fig. 6C). It is important to note that membrane currents are of both polarities and the effective difference between inward and outward currents of a compartment is often essentially smaller than the single components.

**Fig. 7:**
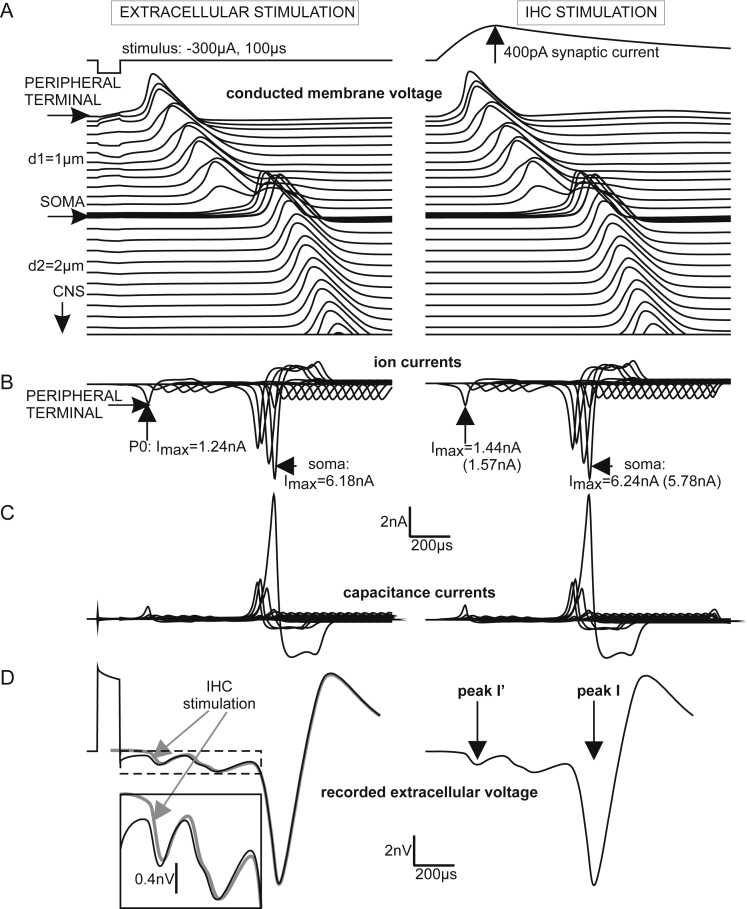
Type I cell responses to extracellular and intracellular stimulation. A: Transmembrane voltages as functions of time are shifted according to the compartment distance along the neural pathway. Striking is the delay when the spike is crossing the soma. B: Sum of ion currents of a compartment (sodium, potassium and leakage current, including synaptic current for P0) is shown as a separate line for each compartment. The first minimum of the right graph (marked by arrow) results from the ion currents of P0 and is the same curve with different scaling from the lowest curve in Fig. 6C. The dominant role of the somatic region is evident. Although the extracellularly applied stimulus has a direct influence on every compartment (comp. voltage profiles during pulse application in A), the ion currents for both stimulation conditions are quite similar, quantified by their *I*_max_ values. The figures in parentheses are the values when peripheral and central axon diameters are enlarged from *d*1 = 1 μm and *d*2 = 2 μm to human average values *d*1 = 1.3 μm and *d*2 = 2.6 μm (Rattay et al., 2013). C: Capacitance currents. The largest values are at the soma followed by the three presomatic non-myelinated compartments. D: Impact of the transmembrane currents from this single spiral ganglion cell to a compound action potential recorded in 2 mm distance as defined in Fig. 2C. The remarkably small difference between extracellular (black) and inner hair cell stimulation (gray) is clearly shown in the insert.

**Fig. 8:**
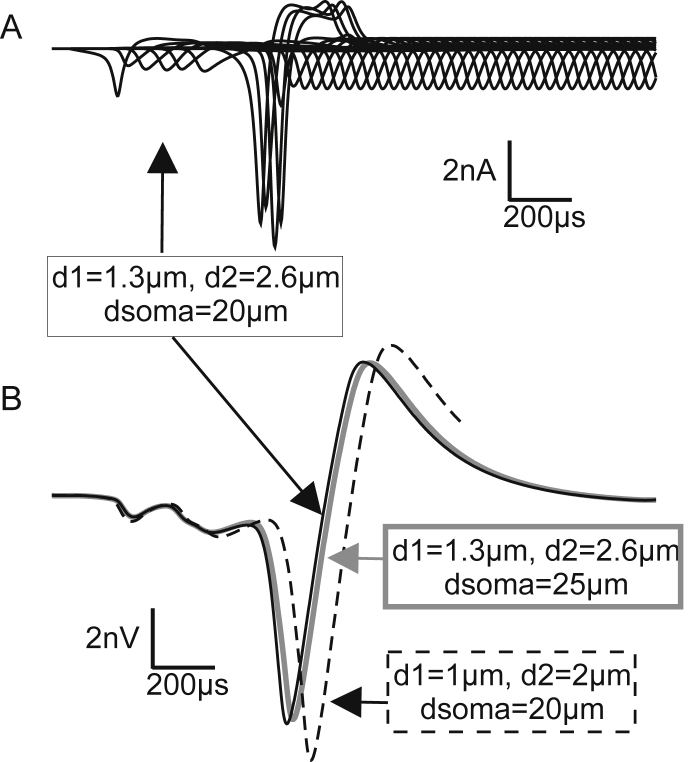
Impact of diameter variation on the potential at the recording electrode for inner hair cell stimulation. A: Ion currents for thicker axons. B: Thicker axons cause quicker signaling but a similar recorded voltage profile. Dashed lines correspond to standard values used in Fig. 7.

The next investigation was a simplified approach to calculate the ABR signal by assuming a recording electrode relatively close to the peripheral SGC section. This electrode position is shown in the right part of Fig. 2C, that is 2 mm in orthogonal direction of the midmodiolar plane which contains the neural path of a target SGC and also a small spherical stimulating electrode. The stimulating electrode was not as close to the cochlear axis as usual with cochlear implants, where it should be as near as possible to the neural structures. The selected position of the stimulating electrode favors spike initiation of the peripheral ending quite similar to natural excitation. A −300 μA square pulse of 100 μs initiated a propagating spike at P0 a bit later than the 400 pA injected current (Fig. 7A) simulating synaptic IHC excitation. In both cases the conducted AP was delayed in the non-myelinated somatic region because of its high capacitance (Rattay et al., 2001a) before being conducted along the thicker central axon with the doubled velocity of the peripheral process (Rattay et al., 2013). The high capacitance of the soma demands enlarged sodium current influx in the presomatic region which consists of three compartments in order to achieve numerical accuracy. The high ion channel densities of these three presomatic compartments together with the low channel density within the large soma surface (compare Fig. 2A) made this region the effective section for the ionic and the capacitance currents (Fig. 7B and C). The simulated membrane currents in the nodes and internodes of the central axon were exactly of doubled intensity compared to that of the peripheral axon when the number of insulating sheets, internodal length and fiber diameter is doubled. From morphometric analysis we did in fact find a constant ratio of 2 for human central/peripheral axon diameter whereas the feline ratio was 1.8 (Rattay et al., 2013). As expected, the model evaluations showed only small deviations from the theoretical prediction that membrane currents and spike velocity increase linear with the diameter of myelinated axons. The predicted velocity ratio of 2:1 was marginally distorted by irregularities at the endings of the rather short peripheral axon. Although axonal membrane currents were of doubled amplitude in the central axon their impact was too small to cause ABR peak I. Evaluation of Eq. (5) demonstrated that the largest ABR wave I contribution of the simulated type I cell resulted from its soma region, whereas no part of the peripheral process had a substantial effect (Fig. 7D). Enlargement of axon or soma diameters had minor influence on the shape of the recorded signal (Fig. 8).

### 3.3 Type I cells with demyelinated peripheral processes, passive soma membrane

**Fig. 9:**
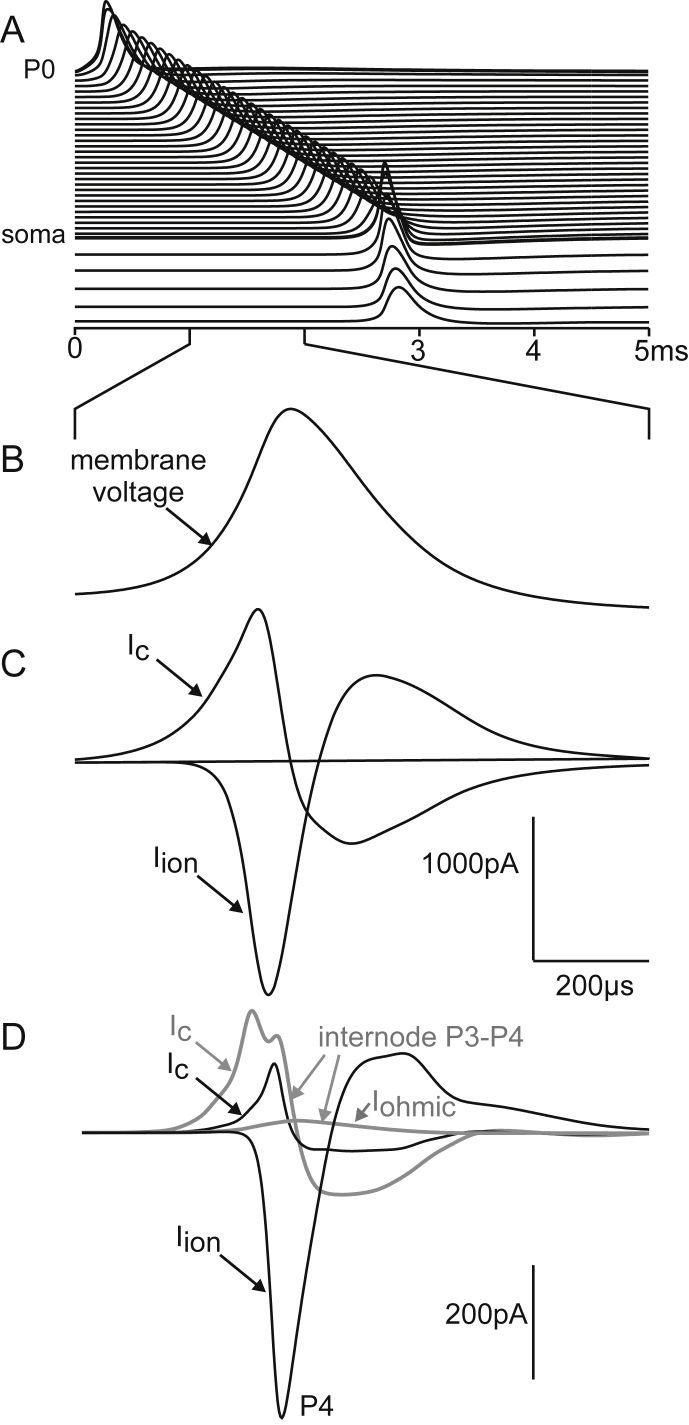
Type I cell with demyelinated peripheral axon. A: Replacement of the myelinated peripheral axon and presomatic segment by a homogeneous fiber with nonmyelinated membrane of original Hodgkin-Huxley ion cannel densities causes slow spike propagation (comp. Fig. 7A). B: Transmembrane voltage of a central compartment; time axis zoomed to 1 ms. C: Transmembrane currents are essentially stronger in the unmyelinated fiber. D: Redrawn currents from Fig. 5B. The compartment length of a node-internode combination of (D) is represented by 5 compartments in (A–C). This factor 2.5 additionally reinforces the effect of current intensity already expressed by different scaling in (C) and (D).

**Fig. 10:**
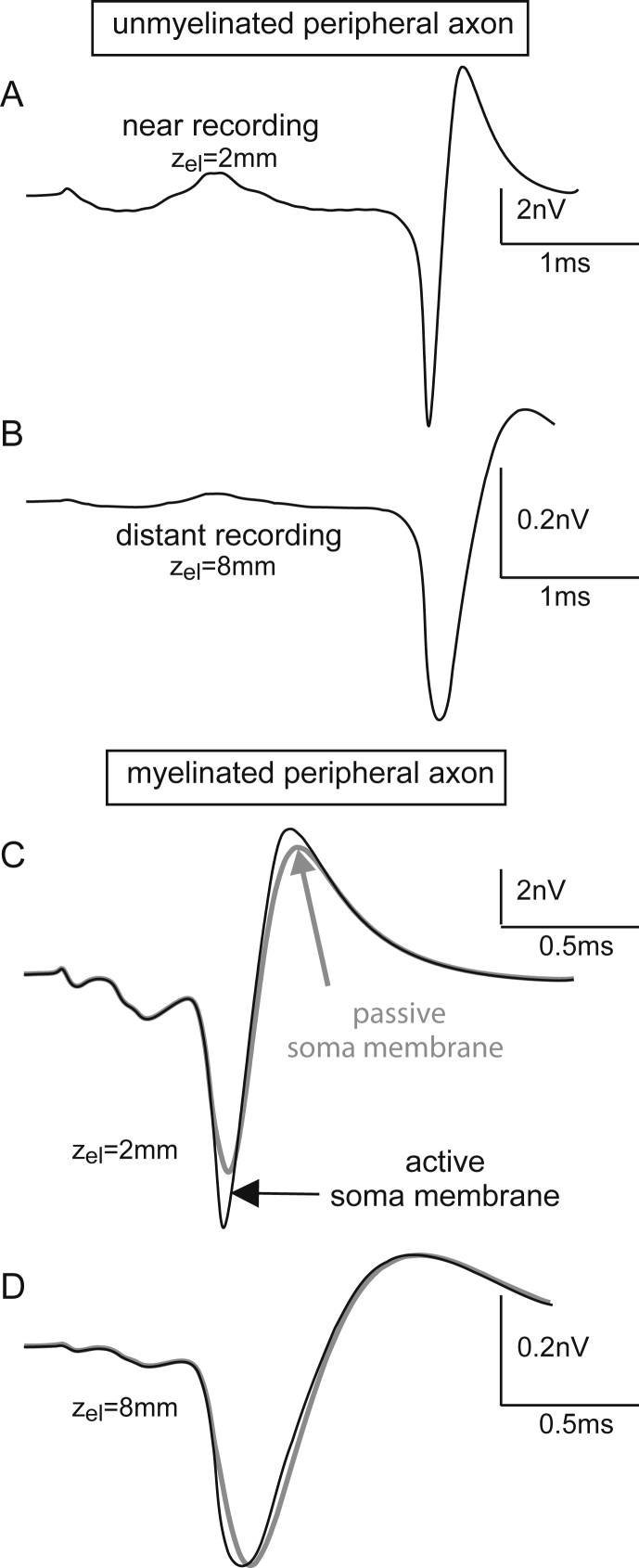
Influence of myelin and active ion channels on the recorded signal. A and B: Recorded responses of a type I cell with unmyelinated peripheral axon, simulated as in Fig. 7. Position of the recording electrode as in Fig. 2C with electrode distance *z*_el_ = 2 mm (A). Increase of electrode distance to *z*_el_ = 8 mm causes a smoothed signal of smaller amplitude (B). C and D: Comparison of the early part of the simulated ABR signal for active soma membrane (black) and passive soma membrane (gray).

The next computer experiment was used to analyze the contribution of unmyelinated type II cells on ABR wave I. For this purpose the peripheral process of the standard SGC was assumed to be unmyelinated and the region from P0 to the soma was modeled as homogeneous membrane with original Hodgkin-Huxley ion channel densities. The demyelination had three essential consequences: (i) AP conduction was slowed down to 23% (compare Fig. 7, Fig. 9A). (ii) Both types of transmembrane currents of an average compartment were more similar in shape and intensity than those of the node-internode combination where the ion current dominated in the node and the capacitance current in the internode (Fig. 9C and D). (iii) Transmembrane current strengths were larger in a section, which was assumed earlier to be myelinated, as the total number of ion channels in this section increased by a large extent. Nevertheless, the increased currents of the peripheral axon could not contribute as much as the soma region to the extracellularly recorded potential. The characteristic somatic contribution was even more obvious when the electrode distance was increased from 2 mm to 8 mm (Fig. 10A and B).

Using a passive membrane to simulate the soma, i.e., excluding the nonlinear gating of ion channels, caused some reduction of the amplitude of the recorded signal in the near recording case (Fig. 10C) but resulted in an almost identical voltage profile for active and passive soma membranes for distant recording (Fig. 10D).

### 3.4 Evaluation with the head model

**Fig. 11:**
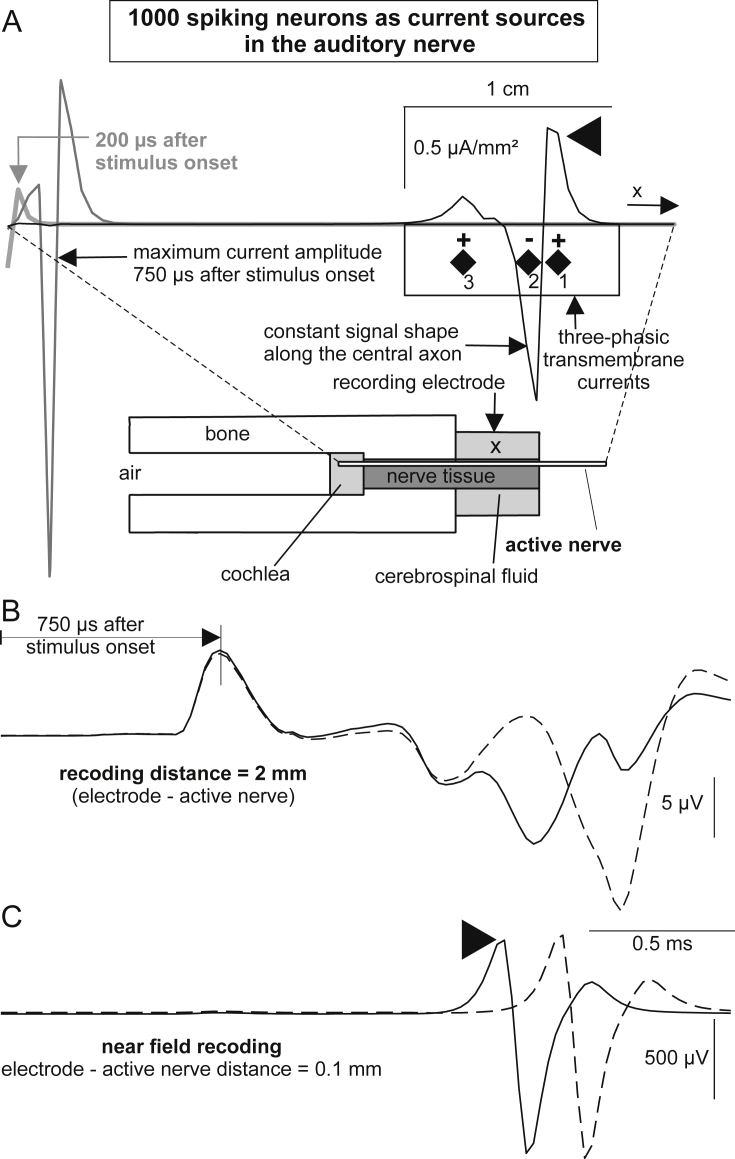
Compound action potentials simulated with the head model. A: The spatial distribution of the current sources along the active cylinder is biphasic in the beginning and three-phasic thereafter. The three-phasic transmembrane currents can be simulated by 2 dipoles defined by the half-wave gravity points 1–2 and 2–3. Lower diagram corresponds to Fig. 4B. B: Recorded signal at position X (full line) and 3 mm further in *x*-direction (dashed line). In both cases CAP peak I coincides exactly with the time (*t* = 750 μs) when transmembrane currents reach their maximum amplitude. C: Near field response, as in B, but electrode is only 0.1 mm from the surface of the active nerve segment.

To emphasize the conclusions about the dominant role of SGC somas as generators of human ABR peak I, the transmembrane currents from a bundle of 1000 synchronously firing type I cells were evaluated with a simplified head model (Fig. 4). Input data were the currents from 64, 0.5 mm long segments that represented the total temporal current flux along the auditory nerve. Each segment comprised the sum of all implicated ion and capacitance currents resulting from at least one node and one internode. Three examples of these driving currents are shown in Fig. 11A for *t* = 200 μs, *t* = 750 μs and *t* = 2200 μs, representing times after stimulus onset when the spike is in the peripheral axon, in the soma region and in the central axon close to brainstem, respectively. Note the biphasic shape at the beginning, which then changes to three phases. About 300 μs after the somatic spike passage, all somatic transmembrane currents vanished (Fig. 7B and C) and the spatial shape of the central axon currents was the same as seen for *t* = 2200 μs in Fig. 11A. A simulated recording (2 mm from the surface of the active cylinder) and the near field response (0.1 mm from the surface of the active cylinder) are shown in Fig. 11B and C for electrode positions as indicated by an ‘x’ in the geometrical sketch in Fig. 11A. In the near field transmembrane currents of the bypassing spikes dominated and caused the shape of the recorded signal (as function of time; Fig. 11C) to be similar to the mirrored picture of the transmembrane currents (as functions of space; Fig. 11A see arrowheads). Moving the recording electrode in constant distance along the active cylinder caused the same three-phasic signal but with a delay corresponding to the traveling time of the spikes. For example, moving the electrode 3 mm to the right caused a delayed near field response (dashed line in Fig. 11C). The recorded signal at 2 mm distance showed a much weaker but broader version of the near field response, which is disturbed differently by far field components when the electrode was again moved by 3 mm (Fig. 11B). Note, however, the constant time of peak I (750 μs after stimulus onset) for different electrode positions.

**Fig. 12:**
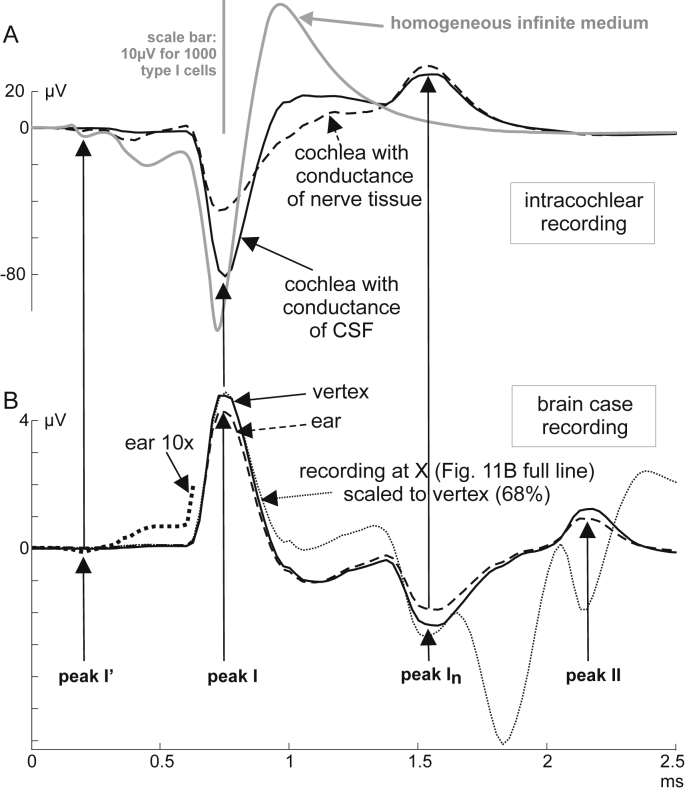
Simulated intracochlear (A) and brain case recordings behind the ear and at the vertex (B) demonstrate synchrony of peak I. Peak I time is constant when cochlear conductance is changed (A) as well as for the 3 recording sites (B). Signals change polarity for intra- and extra cochlear recording. However, in this simulation the polarity for peak I′ is the same when recorded in the cochlea and behind the ear (enlarged curve in B).

Fig. 12A shows simulated intracochlear potentials under the following three conditions: (i) the quite realistic nerve geometry of Fig. 2 but a homogeneous infinite medium, (ii) FE solution for rectified SGCs in an active cylinder and assuming the CSF conductance value within the cochlea, (iii) same situation as in (ii) but the cochlea was simulated with the conductance of nerve tissue. In case (i) the peripheral axon was close to the recording electrode and consequently the first membrane currents caused stronger recorded amplitudes. In all three cases the strongest parts of the recorded signals appear nearly at the same time, i.e., when the spike crosses the soma region as shown in the previous sections. This synchrony of the first peak demonstrates that it was not generated by a change in the conductance of the extracellular medium but it appeared just at the time when the transmembrane currents reach their maximum values. Furthermore, in synchrony with the first negative intracochlear peak a positive peak could be recorded at the vertex and behind the ear (Fig. 12B).

In contrast to the infinite model, the bony boundary of the skull hinders currents to leave the head surface. This was without impact on peak I time but caused about 5 times larger recorded potentials in the head model (Fig. 12A).

The almost synchronous initiation of a high number of spikes and their conduction in the auditory nerve cause a spatial-temporal pattern of currents within the head. At a fixed time after stimulus onset, e.g. at the time of peak I’, the electrical situation in the head and on its surface is characterized by isopotentials. Of specific interest is the isopotential equal zero because this time dependent surface is the border where the polarity changes. In our simple head model peak I’ has the same polarity behind the ear as within the cochlea (magnified doted curve in Fig. 12B versus gray and dashed lines in Fig. 12A), whereas at the time of peak I recording electrodes at vertex or ear are separated by the isopotential 0 from the intracochlear region. Such isopotential lines (on the surface of the head) including isopotential zero are reported systematically for all human ABR peaks I′–VII (Hughes and Fino, 1985).

## 4 Discussion

The phrase ‘auditory brainstem response’ implies the assumption that all its constituent waves may be initiated in the brainstem. Many studies in animals and clinical studies in humans were needed to improve, step by step, the knowledge of the individual ABR peak generators. Disagreements about the peak generators were caused by technical limitations such as lack of modern microelectrodes, ethical considerations for human experiments and confusing observations, e.g. along the pathway of the auditory nerve signal polarity changes at the proximal end of internal auditory meatus. According to this signal polarity effect, ABR pioneer Jewett using letters P and N for positive and negative peaks claimed in his 1970 paper: “With the tongue as a reference point in the deeply anesthetized cat, recordings from the scalp or rostral brain locations obtained by repeated averaging, time-locked to an auditory click, showed four positive waves which were labeled P1–P4. P1 occurs simultaneously with N1 recorded at the round window and is probably generated by the VIIIth nerve”. Several investigators found convincing arguments that also P2 is generated by the auditory nerve when volleys of spikes in afferent fibers enter the brainstem (Moller, 1983, Moller et al., 1988, Martin et al., 1995, Brown and Patuzzi, 2010). Although it is well accepted that stationary components in far field potentials arise when nerve signals cross the border between media with quite different electrical conductances (Stegeman et al., 1987, Martin et al., 1995, Brown and Patuzzi, 2010) hypotheses about where ABR peaks P1 and N1 are generated are controversial. Moreover, it is not clear which of the ABR peaks I′–VII are stationary potentials.

Using sophisticated magnetic resonance imaging techniques, it is possible to map electric conductance of all brain regions (Tuch et al., 2001). Combined with finite element modeling such an approach could predict details of ABR components, even in a patient specific manner. However, including this method is beyond the scope of the presented study. Here, we used a simplified human head model in order to analyze which of the early ABR peaks are stationary potentials. This analysis is based on our recent investigation on spike conduction in SGCs (Rattay et al., 2013), where we found nearly the same velocity–diameter relationships for myelinated SGC axons as recorded in afferent peripheral mammalian axons (Boyd and Kalu, 1979). The 32.35 mm neural path length for the simulated spike conduction was an average value of medical imaging data measured from the peripheral terminals of SGCs to their entry points at the border to the brainstem. Due to the similar conductances of the bone and dura mater a single compartment comprising of both media was used.

The simulated conducted spikes generate ABR N1 when crossing the (bone + dura mater)/CSF border and ABR P2 when arriving at the CSF/brainstem interface (Fig. 12). These results are in agreement with Martin et al. (1995). According to our simulations peak I is generated in the soma region within the cochlea and not at the proximal end of the IAM as supposed by Brown and Patuzzi (2010). They substituted the propagating AP in the axon by two dipoles, which may generate P1 and N1 when each of the dipoles “crosses” the dura mater. According to this hypothesis latency difference N1–P1 should be the same as the arrival time difference of the two dipoles at the conductance barrier. In our computational model the temporal dipole distance is about 0.2 ms. This latency difference is calculated from the three gravity points of the half-waves of the three-phasic curve (full line) seen in Fig. 11C. The first dipole is represented by gravity points 1 and 2, the second by gravity points 2 and 3. Even a bit longer AP duration would cause a much shorter dipole latency difference as recorded in human. Furthermore, the interpretation of Brown and Patuzzi (2010) is rooted in the assumption that “each primary afferent neuron passes through an individual aperture in the highly electrically resistive dura mater sheath”. Yet, at least in human, the dura mater and the arachnoidal membrane invaginate into the IAM from the porus to the fundus (Lescanne et al., 2002) and at the transition zone between peripheral and central nervous system the nerve fibers cross the glia limitans not the dura mater (Fraher, 2002), a glial barrier of much lower thickness than the dura. Thus, the dura mater and the glia limitans are unlikely to be causative for stationary potentials. Rather, the dura and the temporal bone, due to their comparable conductances, electrically form a single macroscopic compartment. Furthermore, even though a tripole, generated by the AP, passes a conductance barrier an unipolar stationary potential is generated (Stegeman et al., 1987). Thus, a single barrier alone cannot be the cause of the successive peaks I and I_n_ as suggested by Brown and Patuzzi (2010).

Reported latency differences N1–P1 of 2.60-1.84 = 0.76 ms (Table 1 of Martin et al., 1995) and 2.32 − 1.67 = 0.65 ms (Table 3 of Hughes and Fino, 1985) are, however, rather close to our results of 0.80 ms (Fig. 12). The same authors found latency differences of 1.32 ms and 1.30 ms for P2–P1, whereas our value was 1.42 ms (Fig. 12). This discrepancy may mainly be caused by morphometric uncertainties about auditory nerve segment lengths in IAM and CSF.

Some authors could record the early ABR peak I’, which is assumed to result from postsynaptic currents of IHCs in type I SGCs (Hughes and Fino, 1985, Moore et al., 1992, Davis-Gunter et al., 2001, Grant et al., 2010, Rattay et al., 2013, Lichtenhan et al., 2014). Our finite element approach was not detailed enough to see these signals in the simulated ABR but we could clearly see it in the intra-cochlear model, e.g. marked by vertical arrows in Fig. 7D, as a first negative peak in Fig. 8B or in Fig. 12. Our biophysical model approach underlines the assumption that the recorded peak I’ is caused by the surprisingly strong postsynaptic currents (Fig. 12; Dolan et al., 1989, Rattay et al., 2013). Recorded latency data I–I′ of 570 μs (left ears) and 620 μs (right ears; Hughes and Fino, 1985) are close to our intra-cochlear 580 μs latency (Fig. 7).

These corresponding latencies I–I′ and the observations that N1 recorded as a compound action potential (CAP) of the auditory nerve at the round window appears simultaneously with ABR P1 (Jewett, 1970, Gersdorff, 1982, Moller et al., 1988) underline that waves I and I’ are not stationary potentials. Moreover, intra-cochlear recordings with cochlear implants showed slightly earlier N1 peaks than CAP recordings at the round window (Miller et al., 2004). As non-human data are obtained from small animals, where commonly wave II was not seen, it was hypothesized that they may have other peak generators as humans, eventually because the internal auditory meatus is essentially longer in man (Moller et al., 1988). Regarding wave II this hypothesis was disproved as the wave II peak was recently shown to be generated as stationary potential in guinea pig (Brown and Patuzzi, 2010). Concerning intracochlearly recorded N1 our single spiral ganglion cell simulation had quite similar temporal properties as found from cat experiments (Fig. 3). According to this simulation, the first part of the central axon was the wave I generator. Contrary to cat, in humans the strong transmembrane currents in the nonmyelinated soma region is seen to generate the first ABR responses.

The non-myelinated type II cells are innervated by OHCs. The smaller number of type II cells is compensated by large transmembrane currents typical for non-myelinated axons. However, for three reasons they cannot rival type I cells concerning ABR peak I generation. First, AP duration is assumed to be the same for both cell types and a dipole model is used where the total transmembrane currents are reduced to two spatially separated currents. In contrast to type I cells, the slow spike conduction velocity of non-myelinated axons causes a short dipole length. A short dipole is not efficient at a distant recording site. Second, the postsynaptic currents from OHC ribbon synapses occur relatively rarely and at 26 pA they are strikingly smaller than the 400 pA amplitudes from IHCs (Grant et al., 2010, Weisz et al., 2012). Third, type II cells do not fire synchronously. The simulated response to the weak stimulus created a postsynaptic delay in the order of 800 μs for spike generation in type II cells (Rattay et al., 2013), which together with the slow AP conduction, hinders an early contribution of the capacitance currents from somas of type II cells.

Our analysis showed that the transmembrane currents of the peripheral axons are rather small (Fig. 6B and C) and they cannot be the generators of ABR peak I. Further evidence for this argument comes from eABR in cochlear implantees. As the electrical stimulating signals are several orders larger than the recorded ones, the first part of the neural response vanishes in the stimulus artifact. However, the latency of peak III can be measured accurately. The value of 2.1 ms for the eABR peak III latency (Starr and Brackmann, 1979) correspond almost exactly with the 2.13 ± 0.14 ms latency difference between peaks III and I as recorded in normal hearing subjects (Esteves et al., 2009). Similar short eABR peak III delays are reported (Guiraud et al., 2007). In contrast to acoustically evoked auditory nerve excitations electrical stimulation causes no delay by mechanical components or by the synaptic processes at the hair cells as the electric field directly stimulates elements of the auditory nerve.

In most cases spikes elicited by human cochlear implants are expected to be initiated in the central axons and not at the peripheral SGC ending as in Fig. 6A where the electrode is quite far from the modiolus. Moving the electrode closer to the modiolus favors more central SGC segments to be stimulated (Rattay et al., 2001a, Rattay et al., 2001b, Potrusil et al., 2012). It is important to note that in people with hearing loss most of the peripheral axons degenerate whereas much more central axons seem to be intact and excitable (Felix et al., 1997, Nadol, 1997). Because of the rather long peripheral human axons, usually the stimulating electrode is distal to the soma and for such cases a theoretical analysis predicts that SGC without peripheral processes cannot be excited with cathodic pulses (Rattay, 1999, Rattay et al., 2001a, Rattay et al., 2001b). This effect was confirmed by ABR and CAP analysis from cochlear implantees showing that the anodic part of biphasic pulses is more effective for excitation (Macherey et al., 2008). This result applies whether the cathodic or the anodic pulse is first and for various inter phase gaps (Undurraga et al., 2013). Therefore, spikes are expected to be initiated in most cases in the distal segments of the central axon in cochlear implantees (Rattay et al., 2001a, Rattay et al., 2001b). These elicited APs generate the large capacitance currents at the somas and the same electrical signaling as in natural hearing when the spikes are conducted along the central axons. Thus the temporal coincidence of 2.1 ms for the eABR peak III latency and the 2.1 ms for the difference between peaks III and I in the healthy ear are strong indicators that the intense soma currents are the generator elements of peak I.

In contrast to the human, nearly all studied mammalian type I cells are myelinated in the soma region in addition to their myelinated axons. Thereby their somatic capacitance is significantly reduced (Rattay, 1990; Rattay et al., 2001a) resulting in a weaker influence of the soma on the ABR signal. However, the example of a feline type I cell (Fig. 2) demonstrates that the transmembrane currents in myelinated soma regions of type I cells may still be strong enough to be the generators of peak I in mammals too.

### 4.1 Model limitations

The investigations were concentrated on the evaluation of the transmembrane currents of single representative type I and type II cells and not on a model that simulates an ABR signal as recorded at the scalp.

A previously presented compartment model for SGCs was used to simulate the transmembrane currents (Rattay et al., 2001a). Important parameters for the choice of the membrane model were the AP duration and its rise time. Different types of voltage-gated ion channels have been identified in SGC membranes based on rat and mouse data (Hossain et al., 2005, Yi et al., 2010, Negm and Bruce, 2008). However, most ion channel models for non-myelinated neural membrane segments in warm blooded animals, including the only available complete model for a SGC (Hossain et al., 2005), show AP durations longer than 1 ms (Hu et al., 2009, Rattay and Wenger, 2010). In contrast to such membrane models, the original Hodgkin-Huxley model with a temperature fit to 27 °C resulted in APs with a duration of 330 μs and a short rise time that was consistent with SGC spikes recorded within the feline cochlea (Fig. 3). During the period of an AP the signal shape for the sum of ion currents is comparable with other models for myelinated peripheral axons but their ion current density amplitudes in the nodes of Ranvier are a bit larger (Rattay and Aberham, 1993, Rattay et al., 2003). Resulting corrections from a more detailed modeling are not expected to contradict the dominant role of the somatic region in generating ABR peak I.
